# Atherogenic Index of Plasma in Relation to Angiographic Coronary Artery Disease Severity, Plasma Fatty Acid Composition, and Estimated Desaturase Activity: A Cross-Sectional Study

**DOI:** 10.3390/metabo16080560

**Published:** 2026-08-08

**Authors:** Saime Batirel, Bengu Cetinkaya, Ali Sahin, Tuba Guctekin, Beste Ozben, Mustafa Kursat Tigen

**Affiliations:** 1 Department of Medical Biochemistry, School of Medicine, Marmara University, Istanbul 34854, Türkiye; 2 Genetic and Metabolic Diseases Research and Investigation Center (GEMHAM), Marmara University, Istanbul 34854, Türkiye; 3 Department of Biochemistry (MED), Institute of Health Science, Marmara University, Istanbul 34854, Türkiye; 4 Department of Cardiology, School of Medicine, Marmara University, Istanbul 34854, Türkiye

**Keywords:** atherogenic index of plasma, triglyceride–glucose index, coronary artery disease, desaturase activity, fatty acid metabolism

## Abstract

**Background/Objectives:** Conventional lipid parameters incompletely reflect coronary atherosclerotic burden, particularly in the setting of metabolic heterogeneity and residual cardiovascular risk. Composite indices such as the atherogenic index of plasma (AIP) and the triglyceride–glucose (TyG) index have emerged as integrative markers. Fatty acid desaturases regulate key pathways of lipid metabolism, and estimated desaturase activity indices (SCD-16, SCD-18, D5D, and D6D) may provide complementary information on the metabolic pathways underlying AIP and the TyG index. However, the relationships among composite atherogenic indices, plasma fatty acid composition, estimated desaturase activity indices, and coronary artery disease (CAD) severity remain incompletely understood. **Methods:** In this cross-sectional study, 61 patients undergoing coronary angiography for suspected CAD were evaluated. Coronary atherosclerotic burden was quantified using the Gensini score. AIP and the TyG index were calculated from fasting biochemical parameters. Plasma fatty acid composition was analyzed using gas chromatography–mass spectrometry, and estimated desaturase activity indices were calculated using product-to-precursor ratios. **Results:** AIP was independently associated with Gensini score (β = 37.020, *p* < 0.001), whereas the TyG index was not independently associated after adjustment for potential confounders (*p* = 0.101). In correlation analyses, AIP was positively correlated with HOMA-IR (ρ = 0.352, *p* < 0.05). Myristic acid was positively correlated with AIP, the TyG index, and VLDL (ρ = 0.297, 0.288, and 0.325, respectively; all *p* < 0.05), whereas n-6 polyunsaturated fatty acids were positively correlated with LDL (ρ = 0.276, *p* = 0.031). Among the estimated desaturase activity indices, D6D was positively correlated with AIP, BMI, insulin, and VLDL (ρ = 0.262, 0.376, 0.340, and 0.266, respectively; all *p* < 0.05), whereas D5D was inversely correlated with BMI and VLDL (ρ = −0.319 and −0.266, respectively; both *p* < 0.05). No significant associations were observed for SCD-16 or SCD-18. **Conclusions:** The observed associations between AIP, angiographic CAD severity, selected fatty acid species, and estimated desaturase activity indices suggest that AIP may provide complementary information regarding metabolic alterations associated with coronary atherosclerosis. These findings provide a basis for future studies exploring the relationship between composite atherogenic indices and fatty acid metabolism.

## 1. Introduction

Coronary artery disease (CAD) remains the leading cause of morbidity and mortality worldwide [1]. Its pathogenesis is driven by atherosclerosis, a chronic process involving lipid accumulation, endothelial dysfunction, vascular inflammation, and insulin resistance [2]. Conventional lipid parameters, including low-density lipoprotein (LDL), high-density lipoprotein (HDL), and triglycerides remain fundamental components of cardiovascular risk assessment [3]. However, they often fail to capture the full spectrum of atherogenic risk, particularly in populations characterized by metabolic heterogeneity. This so-called residual risk paradox, where patients with apparently controlled lipid levels continue to experience coronary events, has prompted increasing interest in integrative biomarkers that better reflect the complex metabolic disturbance underlying atherosclerosis [4]. Composite indices that integrate multiple metabolic dimensions have therefore gained considerable attention.

The Atherogenic Index of Plasma (AIP) has been proposed as a surrogate marker of lipoprotein particle size, small dense LDL prevalence, and the balance between triglyceride-rich lipoproteins and HDL-mediated reverse cholesterol transport, providing a more comprehensive measure of atherogenic dyslipidemia [5,6]. Accordingly, AIP has been shown to be independently associated with CAD presence, angiographic severity, and adverse cardiovascular outcomes [7,8,9].

Triglyceride–Glucose (TyG) index has emerged as a reliable surrogate marker of insulin resistance, a central driver of atherogenesis [10]. However, its relationship with angiographic CAD severity remains inconsistent. While some studies have reported that higher TyG index levels are associated with increased severity of coronary stenosis [11,12], others have failed to demonstrate a significant relationship between TyG index and coronary atherosclerotic burden or plaque characteristics [13,14]. Notably, Wu et al. reported that although the TyG index was associated with CAD presence, it was not related to disease severity [14]. These conflicting findings suggest that TyG index may primarily reflect systemic metabolic dysfunction rather than directly capturing the structural atherosclerotic burden.

Emerging evidence suggests that AIP and TyG index represent distinct but related aspects of cardiometabolic dysfunction, including atherogenic lipid remodeling and insulin resistance-related metabolic disturbances [15]. Although both indices have been associated with cardiovascular risk, their relative relationships with angiographic CAD severity remain incompletely understood. Moreover, the metabolic pathways underlying these associations, particularly those related to fatty acid composition and desaturase-driven lipid remodeling, have not been systematically investigated.

The lipid alterations reflected by AIP and TyG index may be related to metabolic pathways involved in fatty acid metabolism. Among these, stearoyl-CoA desaturase (SCD), delta-5 desaturase (D5D), and delta-6 desaturase (D6D) catalyze key steps in the conversion of saturated fatty acids (SFAs) to monounsaturated (MUFAs) and polyunsaturated fatty acids (PUFAs), thereby influencing membrane composition, triglyceride synthesis, and lipoprotein metabolism [16,17]. Higher D6D activity indices have been associated with insulin resistance, inflammation, and adverse cardiometabolic profiles, whereas lower D5D activity has been linked to obesity, hypertriglyceridemia, insulin resistance, and metabolic syndrome-related phenotypes [18,19]. SCD activity has also been implicated in triglyceride accumulation and VLDL overproduction [16,20]. Despite this evidence, the role of desaturase activity has not been systematically examined in relation to CAD severity, nor integrated with composite atherogenic indices.

The present study aimed to further investigate the relationship between composite atherogenic indices and angiographic CAD severity by integrating plasma fatty acid composition and desaturase activity. Although AIP and TyG index are both established cardiometabolic indices, they reflect distinct yet interconnected aspects of metabolic dysfunction. Therefore, both indices were evaluated simultaneously to better characterize the metabolic pathways underlying coronary atherosclerotic burden. In addition, by integrating free fatty acid composition and desaturase activity indices with AIP and TyG index, we aimed to investigate whether alterations in fatty acid metabolism contribute to the distinct metabolic profiles reflected by these composite indices. Specifically, we sought to determine whether alterations in fatty acid composition and estimated desaturase activity indices are associated with lipid parameters insulin resistance-related metabolic alterations, and angiographic coronary atherosclerotic burden.

## 2. Materials and Methods

### 2.1. Study Design and Participants

This cross-sectional study was designed as an exploratory, hypothesis-generating study and included patients who underwent coronary angiography for the evaluation of suspected CAD between April 2020 and June 2020. The decision to perform coronary angiography was based on the presence of symptoms suggestive of myocardial ischemia and/or documented ischemia on non-invasive testing (exercise electrocardiography, stress echocardiography, or myocardial perfusion imaging). Participants were consecutively recruited from a tertiary care cardiology center to minimize selection bias. The decision to perform coronary angiography was based on standard clinical practice, including symptom assessment, cardiovascular risk profile, and non-invasive diagnostic findings when applicable. 61 participants who met the inclusion criteria were included. Eligible participants were adults undergoing coronary angiography for suspected CAD. Patients with severe systemic diseases such as advanced renal failure, active malignancy, acute infection, inflammatory or autoimmune diseases, severe hepatic disease, recent myocardial infarction, or pregnancy, as well as individuals younger than 18 years were excluded.

Clinical data including age, sex, body mass index (BMI), lifestyle factors, clinical characteristics, and medication use were recorded for all participants. BMI was calculated as weight in kilograms divided by the square of height in meters (kg/m^2^).

All procedures were performed in accordance with the ethical standards of the institutional research committee and the Declaration of Helsinki. The study protocol was approved by the Marmara University School of Medicine Ethics Committee (Approval No: 09.2020.383, approval date: 3 April 2020), and written informed consent was obtained from all participants prior to enrollment.

### 2.2. Coronary Angiography and Assessment of CAD Severity

Coronary angiography was performed using standard techniques via either transfemoral or transradial access with 6–7 French diagnostic catheters, utilizing a commercially available angiography system (Artis Zee biplane; Siemens Healthcare, Erlangen, Germany). The severity and extent of coronary atherosclerosis were assessed using the Gensini scoring system. In briefly, the score was calculated by assigning a severity score to each coronary stenosis according to the degree of luminal narrowing (1 for 25%, 2 for 50%, 4 for 75%, 8 for 90%, 16 for 99%, and 32 for total occlusion). Each lesion score was then multiplied by a segment-specific factor: 5 for the left main coronary artery; 2.5 for the proximal left anterior descending (LAD) and left circumflex (LCx) arteries; 1.5 for the mid LAD; 1 for the apical LAD, first diagonal branch, obtuse marginal branch, distal LCx, all right coronary artery segments, and posterior descending artery; and 0.5 for the second diagonal and posterolateral branches. In left-dominant circulation, factors of 3.5 and 2 were used for the proximal and distal LCx, respectively. The final Gensini score was calculated as the sum of all weighted lesion scores [21]. Patients were categorized according to coronary angiographic findings using the Gensini scoring system. Patients with a Gensini score of 0 were assigned to the CAD (−) group, while those with a Gensini score ≥1 were classified as CAD (+), reflecting the presence of angiographically detectable coronary atherosclerosis.

### 2.3. Biochemical Measurements and Calculation of Atherogenic Indices

Fasting venous blood samples were obtained from all participants prior to the angiographic procedure after an overnight fasting period. Samples were collected into appropriate tubes and processed promptly. Serum and plasma aliquots were stored at −80 °C until analysis. Biochemical measurements, including fasting glucose, insulin, total cholesterol, triglycerides, and HDL, were analyzed using an automated clinical chemistry analyzer (Beckman Coulter AU5800, Beckman Coulter, Inc., Brea, CA, USA). VLDL and LDL levels were derived using standard equations. VLDL was estimated as triglycerides divided by 5, and LDL was calculated as total cholesterol minus HDL and VLDL. Insulin resistance was estimated using the homeostasis model assessment of insulin resistance (HOMA-IR) calculated as: HOMA-IR = [Insulin (µIU/mL) × Glucose (mg/dL)]/405. Subsequently, atherogenic indices were calculated as follows: AIP = log_10_ (triglycerides/HDL), and TyG index = ln (triglycerides × glucose/2).

### 2.4. Fatty Acid Composition and Desaturase Activity Indices

Plasma fatty acid composition was determined using gas chromatography–mass spectrometry (GC–MS). Plasma fatty acids were extracted using the Bligh and Dyer method [22]. The extracted lipids were subsequently converted into fatty acid methyl esters (FAMEs) following the procedure described by Christie et al. [23]. FAMEs were separated and analyzed using gas chromatography–mass spectrometry (GC–MS; QP2010, Shimadzu Corporation, Kyoto, Japan). Chromatographic separation was achieved using a FAMEWAX capillary column (30 m × 0.32 mm internal diameter, 0.25 μm film thickness; Restek, Bellefonte, PA, USA). The ion source and detector temperatures were maintained at 240 °C and 250 °C, respectively. Helium was used as the carrier gas at a constant flow rate of 3 mL/min. The injector temperature was set at 250 °C. The oven temperature program was initiated at 130 °C, increased to 240 °C at a rate of 3 °C/min, and held for 5 min. Samples were injected at a volume of 1 μL with a 1:10 dilution. Fatty acid peaks were identified by comparison with known FAME standards (The Food Industry 37 FAME mix, Restek, Bellefonte, PA, USA).

Individual fatty acids were expressed as percentages of total plasma fatty acids. In addition, total SFAs, MUFAs, PUFAs, n-6 PUFAs, n-3 PUFAs, and the n-6/n-3 PUFA ratio were calculated.

Desaturase enzyme activities were estimated indirectly using product-to-precursor ratios of specific fatty acids. The following indices were calculated: SCD-16, defined as the ratio of palmitoleic acid to palmitic acid, and SCD-18, defined as the ratio of oleic acid to stearic acid. D5D activity was estimated as the ratio of arachidonic acid to di-homo-γ-linolenic acid, while D6D activity was calculated as the ratio of dihomo-γ-linolenic acid to linoleic acid.

### 2.5. Statistical Analysis

Statistical analyses were performed using Jamovi software (version 2.3.26; The jamovi project, Sydney, Australia). The distribution of continuous variables was assessed using the Shapiro–Wilk test. Normally distributed variables were expressed as mean ± standard deviation, whereas non-normally distributed variables were presented as median (interquartile range). Comparisons between groups were performed using the independent samples *t*-test or the Mann–Whitney U test, as appropriate. Categorical variables were expressed as counts and percentages and compared using the chi-square test or Fisher’s exact test when applicable. Correlations between variables were evaluated using Pearson or Spearman correlation coefficients, depending on data distribution. Multivariate linear regression analysis was conducted to determine independent associations between metabolic indices (AIP and TyG index) and CAD severity as assessed by the Gensini score. Models were adjusted for potential confounders, including age, sex, and BMI. Standardized beta coefficients were reported to compare the relative strength of associations. Multicollinearity was assessed using variance inflation factor (VIF) values. A two-tailed *p*-value < 0.05 was considered statistically significant. In addition to statistical significance testing, effect size estimates were calculated to quantify the magnitude of between-group differences. Cohen’s d was reported for variables analyzed using the independent samples *t*-test, rank-biserial correlation for variables analyzed using the Mann–Whitney U test, and Cramer’s V for categorical variables.

## 3. Results

### 3.1. Clinical and Metabolic Phenotyping of the CAD Cohort

The study cohort consisted of 61 patients undergoing coronary angiography, stratified into CAD (+) and CAD (−) groups. Baseline demographic, clinical, lifestyle, and metabolic and medication characteristics of the study population are summarized in Table 1. Sex distribution differed significantly between the CAD (+) and CAD (−) groups. There were no statistically significant differences between the groups in terms of age or body mass index. Similarly, metabolic parameters including fasting glucose, insulin levels, and HOMA-IR did not differ significantly between the groups, suggesting a comparable metabolic background. Lipid profile components, including HDL, LDL, and VLDL levels, were also similar between groups, although LDL showed a non-significant trend toward higher levels in the CAD (+) group. Composite lipid indices, including AIP and TyG index, were numerically higher in patients with CAD, however, these differences did not reach statistical significance. Given the relatively limited number of CAD (−) participants, effect size estimates were additionally calculated to complement *p* values and provide additional information regarding the magnitude of the observed between-group differences. Conventional metabolic variables generally demonstrated small effect sizes, whereas AIP (Cohen’s d = 0.582) showed a moderate effect size and the TyG index (Cohen’s d = 0.476) showed a small-to-moderate effect size despite non-significant *p* values. These results indicate that AIP and the TyG index exhibited larger between-group effect sizes than most other metabolic variables. As expected, the Gensini score was markedly elevated in the CAD (+) group compared to the CAD (−) group, confirming the validity of group stratification based on angiographic disease burden. Detailed data regarding individual fatty acids, fatty acid classes, and estimated desaturase activity indices according to CAD status are provided in Supplementary Table S1.

### 3.2. Associations of Clinical and Metabolic Parameters with Coronary Atherosclerotic Burden

Correlation analyses between clinical/metabolic parameters and Gensini score are presented in Table 2. Among traditional risk factors, HDL demonstrated a significant inverse correlation with Gensini score, indicating that lower HDL levels were associated with increased coronary atherosclerotic burden. In contrast, LDL, VLDL, glucose, insulin, and HOMA-IR were not significantly correlated with Gensini score. Notably, AIP exhibited a significant positive correlation with Gensini score, suggesting that higher atherogenic index values are associated with more severe CAD. Given that HDL is incorporated into the AIP formula, the association between AIP and CAD severity may reflect information captured by the combined triglyceride–HDL ratio rather than HDL alone. The TyG index also showed a positive correlation trend, although this did not reach statistical significance.

**Figure 1:**
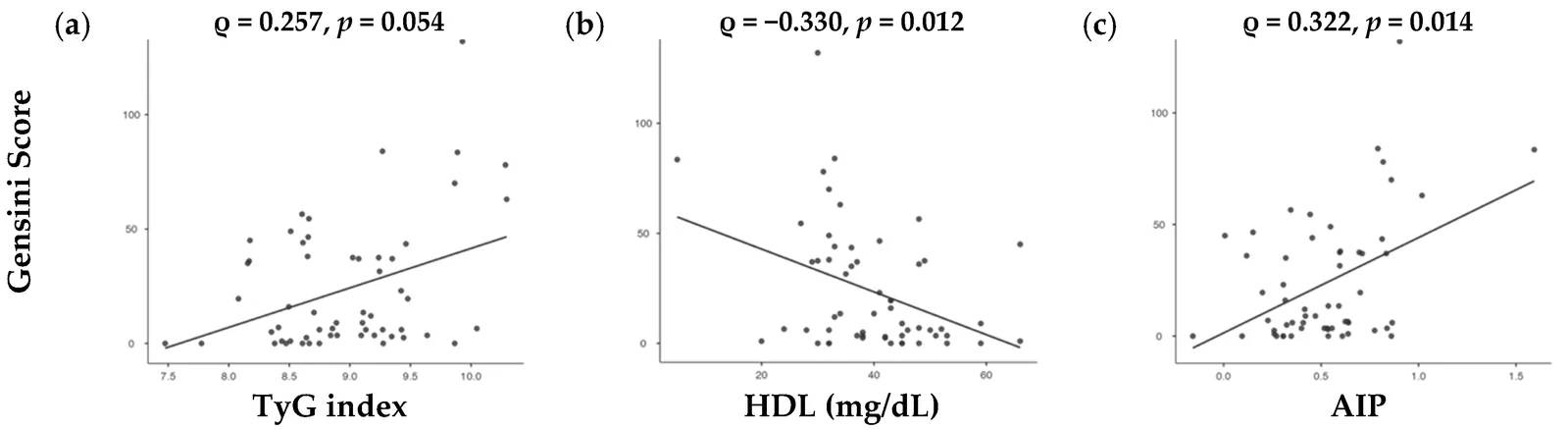
Correlation of lipid parameters with Gensini score. (**a**) The Triglyceride–Glucose (TyG) index exhibited a weaker, non-significant trend. (**b**) HDL demonstrated a significant inverse correlation with Gensini score. (**c**) Atherogenic Index of Plasma (AIP) showed a significant positive association with Gensini score. Solid lines represent linear regression fits with 95% confidence intervals. Grey dots represent individual observations. ρ indicates Spearman’s correlation coefficient.

These relationships are further illustrated in Figure 1, where AIP demonstrated a clear positive association with Gensini score, whereas the relationship between TyG index and Gensini score appeared weaker and more variable.

To identify independent determinants of coronary atherosclerotic burden, multiple linear regression models were constructed using the Gensini score as the dependent variable (Table 3).

In Model 1 (TyG model), which included age, sex, BMI, HDL, and the TyG index, the overall model demonstrated moderate explanatory power (R^2^ = 0.291). Within this model, HDL emerged as a significant independent predictor, while BMI showed a borderline association. The TyG index was not significantly associated with Gensini score, and age and sex were also not significant predictors. These findings indicate that although TyG index showed a positive trend in univariate analysis, it did not retain independent predictive value after adjustment for confounders.

In Model 2 (AIP model), AIP was entered as the lipid-related variable together with age, sex, and BMI. The model fit improved modestly (R^2^ = 0.308). In this model, AIP emerged as an independent predictor of Gensini score, and BMI remained independently associated, while HDL, age, and sex were not significant.

Notably, in Model 3 (HDL model), HDL, which showed a significant inverse correlation with Gensini score in univariate analysis, also remained significantly associated with Gensini score in the HDL-based multivariable model. Because HDL is incorporated into the AIP formula, HDL- and AIP-based lipid representations were evaluated in separate multivariable models with identical covariates to avoid potential mathematical coupling. Although overlap between these measures is expected, the AIP-based model demonstrated modestly improved model fit and retained a significant association with Gensini score. These findings suggest that the triglyceride–HDL relationship represented by AIP may capture aspects of lipid metabolism not fully reflected by HDL alone in relation to angiographic CAD severity.

### 3.3. Associations of AIP and TyG Index with Metabolic Parameters

Following the identification of AIP as an independent predictor of coronary atherosclerotic burden, additional analyses were performed to further characterize the metabolic and lipid components underlying this association. Correlation analyses between AIP, the TyG index, and metabolic and lipid parameters are presented in Table 4.

AIP demonstrated significant positive correlations with insulin and HOMA-IR, indicating a strong association with insulin resistance. The TyG index also showed significant correlations with these parameters.

To further explore these relationships, multivariate linear regression analyses were performed with AIP and the TyG index as dependent variables (Table 5). HOMA-IR remained significantly associated with both AIP and the TyG index, whereas age, sex, and BMI were not significant predictors Although some degree of association is expected because both the TyG index and HOMA-IR incorporate fasting glucose, the persistence of this relationship after adjustment suggests that it may not be explained solely by shared calculation components.

The TyG index showed significant correlations with VLDL and LDL. The association with VLDL is expected, as both variables are strongly dependent on triglyceride levels. Similarly, the relationship with LDL may partly reflect the contribution of triglyceride-rich lipoproteins to LDL metabolism. In contrast, the inverse association with HDL likely reflects underlying metabolic disturbances rather than shared components. As expected, AIP showed strong correlations with triglycerides and calculated VLDL, partly reflecting the triglyceride component shared by these measures.

Among individual fatty acids, myristic acid showed significant positive correlations with both AIP and the TyG index, highlighting its potential role in triglyceride-rich lipoprotein metabolism and atherogenic lipid remodeling. In contrast, arachidic acid showed an inverse association with AIP, indicating that individual saturated fatty acids may exhibit heterogeneous relationships with lipid metabolism.

Taken together, these findings indicate that while both AIP and the TyG index are associated with insulin resistance, AIP more comprehensively reflects lipid remodeling processes and atherogenic dyslipidemia. Therefore, correlations involving composite indices should be interpreted in the context of both their underlying components and the metabolic pathways they represent.

### 3.4. Association of Free Fatty Acids with Lipid Parameters

Given that both AIP and the TyG index are derived from triglyceride- and HDL-related parameters, and considering their observed associations with insulin resistance, additional analyses were performed to explore upstream contributors to lipid remodeling. Correlation analyses between selected free fatty acids and lipoproteins are presented in Table 6. Among fatty acids, lauric acid, a major component of medium-chain fatty acids, demonstrated significant inverse correlations with HDL, whereas linoleic acid showed a positive association with HDL levels. With respect to triglyceride-rich lipoproteins, myristic acid was positively correlated with VLDL levels. In addition, n-6 PUFAs demonstrated a positive correlation with LDL. Taken together, these findings indicate that specific fatty acid species, rather than total fatty acid groups, are differentially associated with lipid parameters. The full correlation matrix is presented in Supplementary Table S2.

### 3.5. Associations of Desaturase Indices with Metabolic and Lipid Parameters

Desaturase activity indices, including SCD-16, SCD-18, D5D, and D6D, were evaluated in relation to metabolic, and lipid parameters (Table 7). None of the desaturase indices showed a significant association with Gensini score or TyG index, whereas D6D was positively correlated with AIP, a composite marker reflecting the balance between triglyceride-rich lipoproteins and HDL.

Among metabolic parameters, D5D was inversely associated with BMI, a marker of adiposity, whereas D6D showed positive correlations with BMI and insulin levels, both reflecting insulin resistance-related metabolic status. In addition, D5D was inversely associated with VLDL, whereas D6D showed a positive association with VLDL, representing triglyceride-rich lipoproteins.

Desaturase indices also showed expected associations with fatty acid groups, including positive correlations of SCD-16 and SCD-18 with MUFAs, inverse associations of SCD-18 and D5D with SFAs, and opposite-direction associations of D5D and D6D with PUFAs, consistent with their respective roles in fatty acid metabolism. Detailed correlations between fatty acid composition and estimated desaturase activity indices are provided in Supplementary Table S3.

Taken together, these findings suggest that fatty acid composition and estimated desaturase activity indices are more closely associated with metabolic and lipid parameters, including AIP, than with angiographic CAD severity itself.

## 4. Discussion

The present study provides an integrated evaluation of the associations among coronary atherosclerotic burden, composite atherogenic indices, fatty acid composition, and estimated fatty acid desaturase activity indices. The principal finding was that the AIP, but not the TyG index, was independently associated with angiographic CAD severity as assessed by the Gensini score. In addition, AIP was associated with specific fatty acid species and estimated desaturase activity indices, particularly D6D. These findings extend current evidence by demonstrating that AIP is associated with both angiographic CAD severity and selected markers of fatty acid metabolism within the same study population.

In the present cohort, traditional cardiovascular risk markers, including LDL, glucose, and insulin, did not differ significantly between CAD (+) and CAD (−) groups. This finding should be interpreted within the context of the present study and the relatively small CAD (−) subgroup and should not be considered as evidence against the established role of these markers in coronary artery disease. To complement the interpretation of these between-group comparisons, we additionally evaluated effect size estimates. The effect size analysis complements the between-group comparisons by suggesting that differences in composite atherogenic indices may be more pronounced than those observed for conventional metabolic markers, despite the absence of statistical significance. When considered together with the correlation and multivariable regression analyses, these findings support the potential value of composite atherogenic indices in the assessment of coronary atherosclerotic burden. Nevertheless, confirmation in larger, more balanced cohorts is warranted. These findings are also consistent with the concept of residual cardiovascular risk, which suggests that conventional lipid and glycemic markers may not fully capture the complexity of atherosclerosis [4]. Previous studies have similarly shown that individual lipid parameters often lack sufficient discriminatory power in contemporary populations, particularly in the presence of metabolic heterogeneity or ongoing treatment [24,25]. These findings further support the concept that static lipid measurements alone may not fully reflect the dynamic metabolic processes associated with coronary atherosclerosis.

Within this framework, AIP showed a significant association with Gensini score and demonstrated modestly improved model fit compared with the HDL-based model. By integrating triglyceride and HDL into a single logarithmic ratio, AIP may capture information derived from both lipid parameters rather than reflecting isolated lipid measures alone [5,25]. Previous studies have similarly reported associations between AIP and both the presence and severity of CAD [8,26]. In the present cohort, although AIP values were not significantly different between CAD (+) and CAD (−) groups, AIP remained independently associated with Gensini score in multivariable analyses and showed modestly improved model fit compared with the HDL-based model. Because HDL is incorporated into the AIP formula, HDL- and AIP-based lipid representations were evaluated in separate multivariable models with identical covariates to avoid potential mathematical coupling. These findings support the concept that HDL may function as part of a broader lipid interaction represented by AIP rather than as an isolated protective factor alone [27].

The findings related to the TyG index provide important clarification in the context of existing literature. Although several studies have reported a positive association be-tween the TyG index and angiographic CAD severity, with higher TyG index levels associated with an increased risk of moderate to severe coronary disease [11,12], these findings have not been consistently replicated across all populations. Indeed, other studies have failed to demonstrate a significant relationship between TyG index and coronary atherosclerotic burden, particularly when assessed using imaging-based methods or in specific subgroups [13]. Similarly, Wu et al. reported that although TyG index was independently associated with the presence of CAD, it was not related to CAD severity, irrespective of glucose metabolic status [14]. In the current study, the TyG index showed a positive but non-significant association with CAD severity in both univariate and multivariate analyses. These findings are consistent with the heterogeneous nature of the existing literature, in which both positive and null associations have been reported [11,12,13,14]. Although no significant association was observed in the present study, the positive trend suggests that the modest sample size may have limited the ability to detect this relationship. Larger studies are needed to clarify this association. This variability may reflect the multifactorial metabolic nature of TyG index, as it is closely linked to both glycemic and lipid parameters, including glucose, insulin, HOMA-IR, VLDL, LDL, and HDL [15]. Taken together, these results support the notion that TyG index may more closely reflect systemic metabolic status rather than directly capturing the structural burden of coronary atherosclerosis in this cohort. Accordingly, TyG index may be better interpreted as a marker of global metabolic risk rather than a direct indicator of angiographic disease severity, positioning it as a complementary, rather than competing, marker alongside AIP [15].

The most novel aspect of the present study is the integration of estimated desaturase activity indices into the evaluation of CAD severity. Desaturase enzymes play central roles in fatty acid metabolism by regulating the conversion of saturated and essential fatty acids into biologically active unsaturated fatty acids, thereby influencing lipid composition, lipoprotein metabolism, and cardiometabolic homeostasis [28,29]. We identified a significant positive association between estimated D6D activity and AIP, suggesting a potential relationship between fatty acid metabolism and atherogenic dyslipidemia. Previous studies have reported that increased D6D activity is associated with adverse metabolic phenotypes, including obesity, insulin resistance, systemic inflammation, and a more atherogenic lipoprotein profile [28]. Furthermore, experimental and epidemiological evidence has linked increased D6D activity to triglyceride-rich lipoprotein metabolism and adverse cardiometabolic profiles [29]. Our findings extend these observations by showing that estimated D6D activity indices were positively associated with AIP in patients with CAD. Taken together, these findings raise the possibility that AIP may reflect broader metabolic alterations related to fatty acid remodeling and desaturase activity. This metabolic shift leads to elevated triglycerides, reduced HDL, and consequently increased AIP. Thus, AIP can be interpreted as a downstream marker of upstream enzymatic dysregulation rather than merely a statistical index. In contrast, D5D activity has been reported to exhibit an inverse relationship with metabolic risk and has been associated with a more favorable lipid and inflammatory profile, highlighting the importance of desaturase balance in cardiometabolic regulation [29]. Consistent with these observations, D5D was inversely associated with BMI, and VLDL levels in our cohort. However, D5D was not significantly associated with either AIP or CAD severity. These findings suggest that D5D may primarily reflect broader metabolic status, whereas D6D appears to be more closely linked to the atherogenic lipid alterations captured by AIP. Previous studies have also linked increased SCD activity to de novo lipogenesis and triglyceride accumulation, contributing to VLDL overproduction and atherogenic dyslipidemia [16]. However, SCD indices were not directly associated with CAD severity in our cohort.

Further support for this metabolic remodeling model is provided by individual fatty acid profiles. Myristic acid was positively correlated not only with AIP and TyG index but also with VLDL, consistent with its reported associations with triglyceride-rich lipoprotein metabolism and atherogenic lipid transport [30]. In addition, n-6 PUFAs showed a positive association with LDL, further supporting the link between fatty acid composition and atherogenic lipoprotein profiles [31]. In contrast, arachidic acid showed an inverse as-sociation with AIP, highlighting the heterogeneity of saturated fatty acids and their differential metabolic effects [32].

Taken together, these findings support an integrated metabolic framework in which fatty acid metabolism, estimated desaturase activity indices, and lipoprotein-related parameters are interconnected. Overall, these findings suggest that composite lipid indices such as AIP may provide additional insight into the relationships between fatty acid metabolism, lipid remodeling, and angiographic coronary atherosclerotic burden.

There are several limitations of this study. First, the cross-sectional design precludes the establishment of causal relationships between metabolic alterations and CAD severity. Second, the present study was designed as an exploratory, hypothesis-generating investigation integrating angiographic assessment with detailed GC–MS-based fatty acid profiling and estimated desaturase activity indices. Therefore, the findings should be interpreted with caution and require validation in larger prospective studies. Third, the relatively modest sample size and the small CAD (−) subgroup may have reduced the precision of between-group comparisons and limited the ability to detect modest associations, such as the relationship between the TyG index and the Gensini score. Therefore, effect size estimates were additionally evaluated to complement statistical significance testing. Because coronary angiography was performed only in clinically indicated patients, inclusion of a larger truly healthy control population was not feasible. Consequently, these findings should be interpreted cautiously and require confirmation in larger, more balanced cohorts. Fourth, patients classified as non-obstructive CAD may still have underlying microvascular dysfunction or early atherosclerotic changes not detectable by conventional angiography. Fifth, desaturase activity was estimated using product-to-precursor ratios rather than direct enzymatic measurements, which may not fully capture in vivo enzyme activity. Sixth, detailed information on dietary intake and physical activity was not comprehensively available, and therefore residual confounding cannot be excluded. Finally, as this was a single-center study, the findings need to be confirmed in a multi-centric study with a larger sample size.

## 5. Conclusions

In conclusion, the present study suggests that AIP is associated with angiographic coronary atherosclerotic burden, retaining its association with Gensini score in the AIP-based multivariable model. While the TyG index provides complementary information related to insulin resistance, its relationship with CAD severity appeared to be more limited and context-dependent in this cohort. In addition, alterations in estimated desaturase activity indices, particularly D6D, were associated with AIP, suggesting a potential relationship between fatty acid metabolism and atherogenic lipid profiles linked to coronary atherosclerotic burden. Collectively, these findings suggest that alterations in fatty acid metabolism and atherogenic lipid remodeling may contribute to the metabolic heterogeneity associated with coronary atherosclerotic burden. Further validation in larger prospective cohorts incorporating established cardiac biomarkers may help clarify the potential clinical utility of integrating composite atherogenic indices with fatty acid metabolism related markers.

## Figures and Tables

**Table 1:** Baseline Clinical and Metabolic Characteristics of the Study Population According to Coronary Artery Disease Status.

	Total	CAD (−) Group (n = 9)	CAD (+) (n = 52)	*p*	Effect Size
Sex (male/female)	34/27	1/8	33/19	**0.004**	V = 0.374
Age (years)	60 ± 12	56 ± 14	61 ± 11	0.227	d = 0.441
Lifestyle factors					
Smoking (%)	31%	22%	33%	0.531	V = 0.080
Alcohol consumption (%)	15%	11%	15%	0.739	V = 0.043
Clinical conditions					
Hypertension (%)	75%	67%	77%	0.509	V = 0.085
Diabetes (%)	49%	22%	54%	0.080	V = 0.224
Medications					
Anti-diabetic (%)	31%	11%	35%	0.160	V = 0.180
Anti-platelet (%)	77%	89%	94%	0.550	V = 0.077
Anti-hypertensive (%)	59%	44%	62%	0.336	V = 0.123
Statins (%)	90%	89%	90%	0.889	V = 0.018
BMI (kg/m^2^)	30.82 ± 6.31	29.11 ± 6.88	31.14 ± 6.22	0.381	d = 0.321
Insulin (mIU/L)	11.39 (11.45)	9.17 (7.9)	11.79 (11.63)	0.668	r = 0.093
Glucose (mg/dL)	116 (51)	114 (69)	116 (47)	0.692	r = 0.086
HOMA-IR	3.45 (3.90)	3.26 (3.11)	3.66 (3.98)	0.483	r = 0.150
Total cholesterol (mg/dL)	183.56 ± 50.86	166.67 ± 31.01	186.48 ± 53.23	0.284	d = 0.390
Triglyceride (mg/dL)	127 (106)	100 (50)	131 (109.5)	0.173	r = 0.289
VLDL (mg/dL)	25.40 (21.20)	20.00 (10.00)	26.20 (21.90)	0.173	r = 0.289
HDL (mg/dL)	39.18 ± 11.01	42.78 ± 10.39	38.56 ± 11.09	0.292	d = −0.384
LDL (mg/dL)	108 (56)	93.00 (30.00)	111.50 (62.50)	0.074	r = 0.229
Gensini Score	9 (34)	0 (3.5)	17.75 (37.88)	**<0.001**	r = 0.773
AIP	0.52 ± 0.28	0.38 ± 0.27	0.55 ± 0.28	0.112	d = 0.582
TyG Index	8.96 ±0.59	8.72 ± 0.60	9.00 ± 0.59	0.193	d = 0.476

Data are presented as mean ± standard deviation (SD) or median (interquartile range, IQR), as appropriate. Categorical variables are presented as counts (n). *p* values < 0.05 were considered statistically significant and are indicated in bold. AIP, atherogenic index of plasma; BMI, body mass index; CAD, coronary artery disease; d, Cohen’s d; HDL, high-density lipoprotein; HOMA-IR, homeostatic model assessment of insulin resistance; LDL, low-density lipoprotein; r, rank-biserial correlation; TyG, triglyceride–glucose; V, Cramer’s V; VLDL, very-low-density lipoprotein.

**Table 2:** Correlation of Clinical and Metabolic Parameters with Gensini Score.

	Gensini Score	
	Spearman’s Rho	*p*
Age (year)	0.166	0.217
BMI (kg/m^2^)	−0.201	0.149
Insulin (mIU/L)	0.043	0.758
Glucose (mg/dL)	0.199	0.137
HOMA-IR	0.086	0.527
Total cholesterol (mg/dL)	−0.107	0.427
Triglyceride (mg/dL)	0.217	0.105
VLDL (mg/dL)	0.217	0.105
HDL (mg/dL)	−0.330	**0.012**
LDL (mg/dL)	−0.157	0.243
AIP	0.322	**0.014**
TyG index	0.257	0.054

Data are presented as Spearman correlation coefficients (ρ). All *p* values were two-tailed, and bold values indicate statistical significance (*p* < 0.05). AIP, atherogenic index of plasma; BMI, body mass index; HDL, high-density lipoprotein; HOMA-IR, homeostatic model assessment of insulin resistance; LDL, low-density lipoprotein; TyG, triglyceride–glucose; VLDL, very-low-density lipoprotein.

**Table 3:** Multivariate Linear Regression Models for Determinants of Gensini Score.

	Model 1 (TyG Model) β (SE)	*p*	Model 2 (AIP Model) β (SE)	*p*	Model 3 (HDL Model) β (SE)	*p*
Age (years)	0.189 (0.268)	0.484	0.260 (0.261)	0.325	0.171 (0.273)	0.533
Sex	−9.523 (6.163)	0.129	−10.701 (5.933)	0.078	−8.969 (6.268)	0.159
BMI (kg/m^2^)	−0.960 (0.493)	0.057	−1.117 (0.485)	**0.026**	−0.856 (0.498)	0.092
HDL (mg/dL)	−0.622 (0.297)	**0.042**	-	-	−0.799 (0.283)	**0.007**
TyG index	9.015 (5.384)	0.101			**-**	**-**
AIP	-	-	37.020 (10.365)	**<0.001**	-	-
Model fit (R^2^): Model 1 = 0.291; Model 2 = 0.308; Model 3 = 0.249						

Data are presented as unstandardized regression coefficients (β) with standard errors (SE). Bold values indicate statistical significance (*p* < 0.05). AIP, atherogenic index of plasma; BMI, body mass index; HDL, high-density lipoprotein; TyG, triglyceride–glucose.

**Table 4:** Correlation of AIP and TyG index with Metabolic and Lipid Parameters.

	AIP		TyG Index	
	Spearman’s Rho	*p*	Spearman’s Rho	*p*
BMI (kg/m^2^)	0.243	0.069	0.194	0.149
Insulin (mIU/L)	0.356	**0.006**	0.390	**0.002**
Glucose (mg/dL)	0.248	0.053	0.569	**<0.001**
HOMA-IR	0.352	**0.005**	0.534	**0.001**
Total cholesterol (mg/dL)	0.235	0.068	0.419	**<0.001**
Triglyceride (mg/dL)	0.865	**<0.001**	0.856	**<0.001**
VLDL (mg/dL)	0.865	**<0.001**	0.856	**<0.001**
HDL (mg/dL)	−0.602	**<0.001**	−0.293	**0.022**
LDL (mg/dL)	0.155	0.234	0.301	**0.018**
C14:0 Myristic acid (%)	0.297	**0.020**	0.288	**0.024**
C16:0 Palmitic acid (%)	0.265	**0.039**	0.207	0.110
C16:1 Palmitoleic acid (%)	0.125	0.335	0.078	0.550
C18:0 Stearic acid (%)	−0.050	0.704	−0.044	0.738
C18:1 Oleic acid (%)	0.020	0.878	0.065	0.618
C18:2 Linoleic acid (%)	−0.189	0.144	−0.061	0.638
C20:0 Arachidic acid (%)	−0.318	**0.013**	−0.208	0.108
C20:4 Arachidonic acid (%)	0.020	0.877	0.050	0.699
C20:5 EPA (%)	0.003	0.985	0.012	0.929
C22:0 Behenic acid (%)	−0.250	0.054	−0.227	0.081
C22:6 n-3 DHA (%)	−0.143	0.271	−0.267	**0.037**
C24:0 Lignoceric acid (%)	−0.090	0.490	−0.132	0.311
SFAs (%)	0.165	0.205	0.074	0.572
MUFAs (%)	0.006	0.963	0.019	0.882
PUFAs (%)	−0.170	0.190	−0.053	0.685
n-6 PUFAs (%)	−0.146	0.263	0.012	0.929
n-3 PUFAs (%)	−0.102	0.436	−0.214	0.098
n-6/n-3 PUFAs	−0.038	0.772	0.198	0.127

Data are presented as Spearman correlation coefficients (ρ). All *p* values were two-tailed, and bold values indicate statistical significance (*p* < 0.05). AIP, Atherogenic Index of Plasma; BMI, body mass index; EPA, eicosapentaenoic acid; DHA, docosahexaenoic acid; HDL, high-density lipoprotein; HOMA-IR, homeostatic model assessment of insulin resistance; LDL, low-density lipoprotein; MUFAs, monounsaturated fatty acids; PUFAs, polyunsaturated fatty acids; SFAs, saturated fatty acids; TyG, Triglyceride–Glucose; VLDL, very-low-density lipoprotein.

**Table 5:** Multivariate Linear Regression Models for Determinants of AIP and TyG Index.

	AIP Dependent Model β (SE)	*p*	TyG Index Dependent Model β (SE)	*p*
HOMA-IR	0.023 (0.011)	**0.045**	0.068 (0.023)	**0.004**
Sex	−0.021 (0.075)	0.780	−0.062 (0.153)	0.688
Age (years)	−0.002 (0.003)	0.498	−0.004 (0.007)	0.535
BMI (kg/m^2^)	0.007 (0.006)	0.244	−0.005 (0.012)	0.682
Model fit (R^2^): AIP Model = 0.126; TyG Model = 0.169				

Data are presented as unstandardized regression coefficients (β) with standard errors (SE). Bold values indicate statistical significance (*p* < 0.05). AIP, Atherogenic Index of Plasma; BMI, body mass index; HOMA-IR, homeostasis of insulin resistance; TyG, Triglyceride–Glucose index.

**Table 6:** Correlations of Selected Fatty Acids with Lipoproteins.

	Lipoprotein	Spearman’s Rho	*p*
C12:0 Lauric acid (%)	HDL	−0.294	**0.022**
C18:2 Linoleic acid (%)	HDL	0.265	**0.039**
C14:0 Myristic acid (%)	VLDL	0.325	**0.011**
n-6 PUFAs (%)	LDL	0.276	**0.031**

Data are presented as Spearman correlation coefficients (ρ). Only statistically significant correlations (*p* < 0.05) are shown, and statistically significant *p* values are indicated in bold. HDL, high-density lipoprotein; LDL, low-density lipoprotein; PUFAs, polyunsaturated fatty acids; VLDL, very-low-density lipoprotein.

**Table 7:** Associations of Desaturase Indices with Clinical, Metabolic, and Lipid Parameters.

	SCD-16		SCD-18		D5D		D6D	
	Spearman’s Rho	*p*	Spearman’s Rho	*p*	Spearman’s Rho	*p*	Spearman’s Rho	*p*
Gensini Score	−0.049	0.717	0.100	0.460	−0.056	0.681	0.082	0.543
AIP	0.087	0.504	0.025	0.849	−0.172	0.185	0.262	**0.042**
TyG index	0.050	0.704	0.050	0.703	−0.070	0.591	0.133	0.308
BMI (kg/m^2^)	0.234	0.079	−0.114	0.399	−0.319	**0.015**	0.376	**0.004**
Insulin (mIU/L)	−0.005	0.969	−0.157	0.239	−0.216	0.104	0.340	**0.009**
Glucose (mg/dL)	−0.088	0.501	0.096	0.462	0.215	0.096	−0.100	0.441
HOMA-IR	−0.055	0.671	−0.064	0.625	−0.105	0.423	0.235	0.068
Total cholesterol (mg/dL)	0.048	0.715	0.072	0.583	−0.118	0.366	−0.008	0.952
Triglyceride (mg/dL)	0.143	0.272	0.040	0.760	−0.266	**0.038**	0.266	**0.038**
VLDL (mg/dL)	0.143	0.272	0.040	0.760	−0.266	**0.038**	0.266	**0.038**
HDL (mg/dL)	0.012	0.926	0.041	0.753	−0.001	0.993	−0.196	0.130
LDL (mg/dL)	0.028	0.827	0.027	0.835	−0.094	0.473	−0.033	0.800

Data are presented as Spearman correlation coefficients (ρ). All *p* values were two-tailed, and bold values indicate statistical significance (*p* < 0.05). AIP, Atherogenic Index of Plasma; BMI, body mass index; D5D, delta-5 desaturase; D6D, delta-6 desaturase; HDL, high-density lipoprotein; HOMA-IR, homeostatic model assessment of insulin resistance; LDL, low-density lipoprotein; SCD-16, stearoyl-CoA desaturase-16 index; SCD-18, stearoyl-CoA desaturase-18 index; TyG, triglyceride–glucose; VLDL, very-low-density lipoprotein.

## Data Availability

The datasets used and/or analyzed during the current study are available from the corresponding author upon reasonable request.

## Supplementary Materials

The following supporting information can be downloaded at: https://www.mdpi.com/article/10.3390/metabo16080560/s1, Table S1: Fatty Acid Composition, and Estimated Desaturase Activity Indices According to Coronary Artery Disease Status; Table S2: Correlations of Fatty Acids with Lipoproteins; Table S3: Correlations of Fatty Acids with Desaturase Indices.

## Abbreviations

The following abbreviations are used in this manuscript:

AIP	atherogenic index of plasma
BMI	body mass index
CAD	coronary artery disease
d	Cohen’s d
D5D	delta-5 desaturase
D6D	delta-6 desaturase
DHA	docosahexaenoic acid
EPA	eicosapentaenoic acid
GC–MS	gas chromatography–mass spectrometry
HDL	high-density lipoprotein
HOMA-IR	homeostatic model assessment of insulin resistance
IQR	interquartile range
LAD	left anterior descending
LCx	left circumflex
LDL	low-density lipoprotein
MUFAs	monounsaturated fatty acids
PUFAs	polyunsaturated fatty acids
r	rank-biserial correlation
SCD	stearoyl-CoA desaturase
SCD-16	stearoyl-CoA desaturase-16 index
SCD-18	stearoyl-CoA desaturase-18 index
SD	standard deviation
SFAs	saturated fatty acids
TyG	triglyceride–glucose
V	Cramer’s V
VLDL	very-low-density lipoprotein

## References

[1] Bergmark B.A., Mathenge N., Merlini P.A., Lawrence-Wright M.B., Giugliano R.P. (2022). Acute coronary syndromes. Lancet.

[2] Ormazabal V., Nair S., Elfeky O., Aguayo C., Salomon C., Zuñiga F.A. (2018). Association between insulin resistance and the development of cardiovascular disease. Cardiovasc. Diabetol..

[3] Vrints C., Andreotti F., Koskinas K.C., Rossello X., Adamo M., Ainslie J., Banning A.P., Budaj A., Buechel R.R., Chiariello G.A. (2024). 2024 ESC Guidelines for the management of chronic coronary syndromes. Eur. Heart J..

[4] Matsuura Y., Kanter J.E., Bornfeldt K.E. (2019). Highlighting Residual Atherosclerotic Cardiovascular Disease Risk. Arterioscler. Thromb. Vasc. Biol..

[5] Dobiásová M., Frohlich J. (2001). The plasma parameter log (TG/HDL-C) as an atherogenic index: Correlation with lipoprotein par-ticle size and esterification rate in apoB-lipoprotein-depleted plasma (FER_HDL_). Clin. Biochem..

[6] Dobiásová M. (2006). AIP--atherogenic index of plasma as a significant predictor of cardiovascular risk: From research to practice. Vnitr. Lek..

[7] Wu T.T., Gao Y., Zheng Y.Y., Ma Y.T., Xie X. (2018). Atherogenic index of plasma (AIP): A novel predictive indicator for the coronary artery disease in postmenopausal women. Lipids Health Dis..

[8] Cai G., Liu W., Lv S., Wang X., Guo Y., Yan Z., Du Y., Zhou Y. (2019). Gender-specific associations between atherogenic index of plasma and the presence and severity of acute coronary syndrome in very young adults: A hospital-based observational study. Lipids Health Dis..

[9] Ran X., Wu Z., Guo D., Zhang S., Liu L., Chen S., Song C., Zhang C., Yang J. (2025). Association between the atherogenic index of plasma and major adverse cardiovascular events in patients with premature coronary artery disease. Eur. J. Med. Res..

[10] Tahapary D.L., Pratisthita L.B., Fitri N.A., Marcella C., Wafa S., Kurniawan F., Rizka A., Tarigan T.J.E., Harbuwono D.S., Purnamasari D. (2022). Challenges in the diagnosis of insulin resistance: Focusing on the role of HOMA-IR and Tryglycer-ide/glucose index. Diabetes Metab. Syndr..

[11] Lu X., Lin X., Cai Y., Zhang X., Meng H., Chen W., Yu P., Chen X. (2024). Association of the triglyceride-glucose index with severity of coronary stenosis and in-hospital mortality in patients with acute ST elevation myocardial infarction after percutaneous coronary intervention: A multicentre retrospective analysis cohort study. BMJ Open.

[12] Xu Z., Chen P., Wang L., Yan J., Yan X., Li D. (2024). Relationship between TyG index and the degree of coronary artery lesions in pa-tients with H-type hypertension. Cardiovasc. Diabetol..

[13] Wang X., Ji X., Yu J., Wang F. (2023). Correlation between TyG index and coronary atherosclerosis assessed by CCTA in elderly male patients: A cross-sectional study. Diabetol. Metab. Syndr..

[14] Wu X., Qiu W., Yang H., Chen Y.J., Liu J., Zhao G. (2024). Associations of the triglyceride-glucose index and atherogenic index of plasma with the severity of new-onset coronary artery disease in different glucose metabolic states. Cardiovasc. Diabetol..

[15] Zeng Q., Zhong Q., Zhao L., An Z., Li S. (2024). Combined effect of triglyceride-glucose index and atherogenic index of plasma on cardiovascular disease: A national cohort study. Sci. Rep..

[16] Svendsen K., Olsen T., Nordstrand Rusvik T.C., Ulven S.M., Holven K.B., Retterstøl K., Telle-Hansen V.H. (2020). Fatty acid profile and estimated desaturase activities in whole blood are associated with metabolic health. Lipids Health Dis..

[17] Žák A., Jáchymová M., Burda M., Staňková B., Zeman M., Slabý A., Vecka M., Šeda O. (2022). FADS Polymorphisms Affect the Clinical and Biochemical Phenotypes of Metabolic Syndrome. Metabolites.

[18] Murakami K., Sasaki S., Takahashi Y., Uenishi K., Watanabe T., Kohri T., Yamasaki M., Watanabe R., Baba K., Shibata K. (2008). Lower estimates of delta-5 desaturase and elongase activity are related to ad-verse profiles for several metabolic risk factors in young Japanese women. Nutr. Res..

[19] Kawashima A., Sugawara S., Okita M., Akahane T., Fukui K., Hashiuchi M., Kataoka C., Tsukamoto I. (2009). Plasma fatty acid compo-sition, estimated desaturase activities, and intakes of energy and nutrient in Japanese men with abdominal obesity or metabolic syndrome. J. Nutr. Sci. Vitaminol..

[20] Hua M.C., Su H.M., Lai M.W., Yao T.C., Tsai M.H., Liao S.L., Lai S.H., Huang J.L. (2021). Palmitoleic and Dihomo-gamma-Linolenic Acids Are Positively Associated With Abdominal Obesity and Increased Metabolic Risk in Children. Front. Pediatr..

[21] Gensini G.G. (1983). A more meaningful scoring system for determining the severity of coronary heart disease. Am. J. Cardiol..

[22] Bligh E.G., Dyer W.J. (1959). A rapid method of total lipid extraction and purification. Can. J. Biochem. Physiol..

[23] Christie W.W. (1993). Preparation of ester derivatives of fatty acids for chromatographic analysis. Adv. Lipid Methodol..

[24] Wu J., Zhou Q., Wei Z., Wei J., Cui M. (2021). Atherogenic Index of Plasma and Coronary Artery Disease in the Adult Population: A Meta-Analysis. Front. Cardiovasc. Med..

[25] Zhu X.W., Deng F.Y., Lei S.F. (2015). Meta-analysis of Atherogenic Index of Plasma and other lipid parameters in relation to risk of type 2 diabetes mellitus. Prim. Care Diabetes.

[26] Li Y., Feng Y., Li S., Ma Y., Lin J., Wan J., Zhao M. (2023). The atherogenic index of plasma (AIP) is a predictor for the severity of coronary artery disease. Front. Cardiovasc. Med..

[27] Dobiásová M. (2004). Atherogenic index of plasma [log(triglycerides/HDL-cholesterol)]: Theoretical and practical implications. Clin. Chem..

[28] Do H.J., Chung H.K., Moon J., Shin M.J. (2011). Relationship between the estimates of desaturase activities and cardiometabolic phenotypes in Koreans. J. Clin. Biochem. Nutr..

[29] Warensjö E., Rosell M., Hellenius M.L., Vessby B., De Faire U., Risérus U. (2009). Associations between estimated fatty acid desaturase activities in serum lipids and adipose tissue in humans: Links to obesity and insulin resistance. Lipids Health Dis..

[30] Kang M., Lee A., Yoo H.J., Kim M., Kim M., Shin D.Y., Lee J.H. (2017). Association between increased visceral fat area and alterations in plasma fatty acid profile in overweight subjects: A cross-sectional study. Lipids Health Dis..

[31] Moussavi Javardi M.S., Madani Z., Movahedi A., Karandish M., Abbasi B. (2020). The correlation between dietary fat quality indices and lipid profile with Atherogenic index of plasma in obese and non-obese volunteers: A cross-sectional descriptive-analytic case-control study. Lipids Health Dis..

[32] Khamlaoui W., Mehri S., Hammami S., Hammouda S., Chraeif I., Elosua R., Hammami M. (2020). Association Between Genetic Vari-ants in FADS1-FADS2 and ELOVL2 and Obesity, Lipid Traits, and Fatty Acids in Tunisian Population. Clin. Appl. Thromb. Hemost..

