# Supplementary material for: Atherogenic Index of Plasma in Relation to Angiographic Coronary Artery Disease Severity, Plasma Fatty Acid Composition, and Estimated Desaturase Activity: A Cross-Sectional Study
Source: Metabolites. 2026 Aug 8;16(8):560. doi: 10.3390/metabo16080560 (PMC13515659; doi:10.3390/metabo16080560)
Supplement: Supplementary file 1 [file metabolites-16-00560-s001.zip › metabolites-4443199-supplementary.pdf]

Supplementary Table S1. Fatty Acid Composition, and Estimated Desaturase Activity Indices According to Coronary Artery Disease Status

	Total	CAD (-) Group (n=9)	CAD (+) (n=52)	*p*	Effect size
C14:0 Myristic acid (%)	1.27 ± 0.34	1.10 ± 0.31	1.30 ± 0.34	0.099	d= 0.605
C16:0 Palmitic acid (%)	43.36 ± 2.51	43.48 ± 2.06	43.33 ± 2.60	0.872	d= 0.059
C16:1 Palmitoleic acid (%)	0.68 (0.41)	0.71 (0.36)	0.68 (0.42)	0.522	r= 0.137
C18:0 Stearic acid (%)	18.18 ± 1.98	19.11 ± 2.01	18.02 ± 1.95	0.127	d= 0.559
C18:1 Oleic acid (%)	8.66 ± 1.61	7.99 ± 1.68	8.77 ± 1.58	0.179	d= 0.491
C18:2 Linoleic acid (%)	15.07 ± 2.34	14.89 ± 2.04	15.11 ± 2.40	0.802	d= 0.091
C20:0 Arachidic acid (%)	0.21 (0.05)	0.23 (0.06)	0.21 (0.05)	0.314	r= 0.214
C20:4 Arachidonic acid (%)	4.95 ± 1.11	5.36 ± 1.68	4.88 ± 0.98	0.239	d= 0.430
C20:5 EPA (%)	0.13 (0.11)	0.11 (0.05)	0.14 (0.12)	0.072	r= 0.380
C22:0 Behenic acid (%)	0.42 ± 0.12	0.46 ± 0.13	0.41 ± 0.12	0.211	d= 0.457
C22:6 n-3 DHA (%)	1.80 (0.82)	1.66 (0.48)	1.81 (0.86)	0.784	r= 0.060
C24:0 Lignoceric acid (%)	0.27 (0.14)	0.24 (0.04)	0.28 (0.15)	0.125	r= 0.325
SFAs (%)	65.15 ± 3.01	66.06 ± 2.38	65.00 ± 3.10	0.331	d= - 0.354
MUFAs (%)	10.76 ± 1.96	10.05 ± 1.96	10.88 ± 1.84	0.223	d= 0.444
PUFAs (%)	24.02 ± 2.17	23.89 ± 1.17	24.04 ± 2.31	0.845	d= 0.071
n-6 PUFAs (%)	21.76 ± 2.24	21.88 ± 1.07	21.74 ± 2.39	0.776	d= - 0.062
n-3 PUFAs (%)	2.11 (0.88)	1.91 (0.53)	2.11 (1.01)	0.483	r= 0.150
n-6/n-3 PUFAs	10.33 (4.87)	10.93 (3.58)	10.13 (5.01)	0.483	r= 0.150
D6D Index	0.09 (0.05)	0.09 (0.05)	0.09 (0.04)	0.768	r= 0.064
D5D Index	3.74 (1.54)	3.85(1.58)	3.71 (1.57)	0.324	r= 0.209
SCD-16	0.02 (0.01)	0.02	0.02	0.576	r= 0.120
SCD-18	0.49 ± 0.12	0.43 ± 0.12	0.50 ± 0.12	0.112	d= 0.582

Data are presented as mean ± standard deviation (SD) or median (interquartile range, IQR), as appropriate. Categorical variables are presented as counts (n). CAD, coronary artery disease; D5D, delta-5 desaturase; D6D, delta-6 desaturase; d, Cohen's d; DHA, docosahexaenoic acid; EPA, eicosapentaenoic acid; MUFAs, monounsaturated fatty acids; PUFAs, polyunsaturated fatty acids; r, rank-biserial correlation; SCD-16, stearoyl-CoA desaturase-16 index; SCD-18, stearoyl-CoA desaturase-18 index; SFAs, saturated fatty acids.

Supplementary Table S2. Correlations of Fatty Acids with Lipoproteins.

	HDL		VLDL		LDL	
	Spearman's rho	*p*	Spearman's rho	*p*	Spearman's rho	*p*
C12:0 Lauric acid (%)	- 0.294	**0.022**	0.002	0.989	- 0.197	0.128
C14:0 Myristic acid (%)	- 0.168	0.195	0.325	0.**011**	0.087	0.504
C16:0 Palmitic acid (%)	- 0.155	0.232	0.240	0.063	- 0.055	0.673
C16:1 Palmitoleic acid (%)	- 0.014	0.912	0.171	0.188	0.021	0.875
C18:0 Stearic acid (%)	- 0.043	0.742	- 0.071	0.585	- 0.074	0.568
C18:1 Oleic acid (%)	0.029	0.824	0.031	0.815	- 0.027	0.836
C18:2 Linoleic acid (%)	0.265	**0.039**	- 0.095	0.468	0.235	0.068
C20:0 Arachidic acid (%)	0.242	0.061	- 0.281	**0.028**	- 0.016	0.905
C20:4 Arachidonic acid (%)	- 0.112	0.390	- 0.041	0.756	0.002	0.985
C20:5 EPA (%)	0.068	0.603	0.038	0.771	- 0.036	0.785
C22:0 Behenic acid (%)	0.193	0.137	- 0.220	0.089	0.041	0.755
C22:6 n-3 DHA (%)	- 0.004	0.976	- 0.217	0.093	- 0.159	0.220
C24:0 Lignoceric acid (%)	- 0.038	0.772	- 0.170	0.191	0.081	0.534
SFAs (%)	- 0.184	0.156	0.116	0.375	- 0.126	0.332
MUFAs (%)	0.035	0.790	0.016	0.900	- 0.023	0.861
PUFAs (%)	0.217	0.094	- 0.099	0.449	0.243	0.059
n-6 PUFAs (%)	0.232	0.072	- 0.044	0.739	0.276	**0.031**
n-3 PUFAs (%)	- 0.005	0.969	- 0.164	0.206	- 0.146	0.262
n-6/n-3 PUFAs	0.083	0.524	0.128	0.326	0.219	0.091

Data are presented as Spearman correlation coefficients (ϱ). All p values were two-tailed, and bold values indicate statistical significance ($p < 0.05$). EPA, eicosapentaenoic acid; DHA, docosahexaenoic acid; HDL, high-density lipoprotein; LDL, low-density lipoprotein; MUFAs, monounsaturated fatty acids; PUFAs, polyunsaturated fatty acids; SFAs, saturated fatty acids; VLDL, very-low-density lipoprotein.

Supplementary Table S3. Correlations of Fatty Acids with Desaturase Indices

	SCD-16		SCD-18		D5D		D6D	
	Spearman's rho	*p*	Spearman's rho	*p*	Spearman's rho	*p*	Spearman's rho	*p*
C14:0 Myristic acid (%)	0.385	**0.002**	0.138	0.287	- 0.259	**0.044**	0.308	**0.016**
C16:0 Palmitic acid (%)	0.052	0.689	- 0.236	0.067	- 0.252	0.050	0.285	**0.026**
C16:1 Palmitoleic acid (%)	0.987	**< 0.001**	0.336	**0.008**	- 0.108	0.404	0.334	**0.009**
C18:0 Stearic acid (%)	- 0.383	**0.002**	- 0.800	**< 0.001**	- 0.050	0.699	0.194	0.134
C18:1 Oleic acid (%)	0.307	**0.016**	0.940	**< 0.001**	0.141	0.278	- 0.225	0.082
C18:2 Linoleic acid (%)	- 0.219	0.091	0.185	0.152	0.058	0.654	- 0.707	**< 0.001**
C20:0 Arachidic acid (%)	- 0.471	**< 0.001**	- 0.302	**0.018**	- 0.026	0.844	- 0.187	0.148
C20:4 Arachidonic acid (%)	0.058	0.654	- 0.109	0.402	0.621	**< 0.001**	0.202	0.119
C20:5 EPA (%)	0.224	0.083	0.257	**0.046**	- 0.077	0.556	0.306	**0.017**
C22:0 Behenic acid (%)	- 0.509	**< 0.001**	- 0.424	**< 0.001**	- 0.005	0.972	- 0.126	0.338
C22:6 n-3 DHA (%)	0.311	**0.015**	0.212	0.100	- 0.003	0.983	0.216	0.095
C24:0 Lignoceric acid (%)	- 0.491	**0.001**	- 0.220	0.088	0.154	0.236	- 0.244	0.058
SFAs (%)	- 0.180	0.166	- 0.731	**< 0.001**	- 0.255	**0.047**	0.430	**< 0.001**
MUFAs (%)	0.413	**0.001**	0.927	**< 0.001**	0.109	0.404	- 0.178	0.170
PUFAs (%)	- 0.137	0.290	0.195	0.133	0.265	**0.039**	- 0.477	**< 0.001**
n-6 PUFAs (%)	- 0.177	0.173	0.130	0.318	0.272	**0.035**	- 0.526	**< 0.001**
n-3 PUFAs (%)	0.302	**0.018**	0.278	**0.030**	- 0.029	0.824	0.213	0.100
n-6/n-3 PUFAs	- 0.334	**0.009**	- 0.233	0.070	0.067	0.606	- 0.322	**0.012**

Data are presented as Spearman correlation coefficients (ϱ). All p values were two-tailed, and bold values indicate statistical significance ($p < 0.05$). D5D, delta-5 desaturase; D6D, delta-6 desaturase; DHA, docosahexaenoic acid; EPA, eicosapentaenoic acid; MUFAs, monounsaturated fatty acids; PUFAs, polyunsaturated fatty acids; SCD-16, stearoyl-CoA desaturase-16 index; SCD-18, stearoyl-CoA desaturase-18 index; SFAs, saturated fatty acids.
